# Quinolone-resistant *Campylobacter* Infections: Risk Factors and Clinical Consequences^1^

**DOI:** 10.3201/eid1006.030669

**Published:** 2004-06

**Authors:** Jørgen Engberg, Jakob Neimann, Eva Møller Nielsen, Frank Møller Aarestrup, Vivian Fussing

**Affiliations:** * Statens Serum Institut, Copenhagen, Denmark;; † Herlev University Hospital, Herlev, Denmark;; ‡ Danish Institute for Food and Veterinary Research, Søborg, Denmark;; § Danish Institute for Food and Veterinary Research, Copenhagen, Denmark

**Keywords:** Campylobacter, jejuni, coli, resistance, quinolones, macrolides, typing, comparison, risk

## Abstract

We integrated data on quinolone and macrolide susceptibility patterns with epidemiologic and typing data from *Campylobacter jejuni* and *C. coli* infections in two Danish counties. The mean duration of illness was longer for 86 patients with quinolone-resistant *C. jejuni* infections (median 13.2 days) than for 381 patients with quinolone-sensitive *C. jejuni* infections (median 10.3 days, p = 0.001). Foreign travel, eating fresh poultry other than chicken and turkey, and swimming were associated with increased risk for quinolone-resistant *C. jejuni* infection. Eating fresh chicken (of presumably Danish origin) was associated with a decreased risk. Typing data showed an association between strains from retail food products and broiler chickens and quinolone-sensitive domestically acquired *C. jejuni* infections. An association between treatment with a fluoroquinolone before stool-specimen collection and having a quinolone-resistant *C. jejuni* infection was not observed.

*Campylobacter* is a leading cause of bacterial gastroenteritis in industrialized and developing countries worldwide (*1*). Most *Campylobacter* infections need not be treated with antimicrobial agents. However, in a subset of patients *Campylobacter* may cause severe complications and increased risk for death and therefore requires treatment. A recent Danish study has shown that patients with *Campylobacter* infections have higher acute- and long-term death rates than controls after coexisting conditions were taken into account (*2*). The drug of choice is a macrolide (e.g., erythromycin or a newer agent) for treatment of enteric *Campylobacter* infections after the microbiologic diagnosis. However, for the empiric treatment of adults with suspected bacterial gastroenteritis, the drug of choice typically includes a fluoroquinolone (e.g., ciprofloxacin) because of their activity against almost all enteric bacterial pathogens. Antimicrobial drug resistance in *Campylobacter* infections, in particular to quinolones, has increased dramatically in many countries during the 1990s as reviewed by Engberg et al. (*3*). According to a recent published report by World Health Organization (*4*), the sources of antimicrobial drug–resistant *Campylobacter* strains and the clinical impact of such strains need to be determined.

We conducted a 1-year prospective study to address the prevalence of macrolide and quinolone resistance in human *Campylobacter* isolates. Human isolates were compared with isolates from retail food products and broiler chickens. A systematic approach integrating standardized epidemiologic, antimicrobial susceptibility, and typing data was used. We also conducted a case-comparison study to identify risk factors associated with acquiring quinolone-resistant *C. jejuni* infections.

## Materials and Methods

### Surveillance and Susceptibility Testing of *Campylobacter* Isolates

The study included all culture-positive *Campylobacter* infections from May 1, 2001, through June 10, 2002, from two counties with a catchment area of approximately 1.1 million persons (approximately one fifth of the Danish population). The county of Copenhagen, a metropolitan residential area, has a population of 619,000, and the county of Funen, an island with both urban and rural areas, has a population of 472,000. Because of inconsistencies in the patient enrolment from the county of Funen during May and June 2002, patients from this county who were infected after April 31, 2002, were excluded. Epidemiologic data were captured on self-completed standardized patient questionnaires forwarded by the Danish Zoonosis Centre. Patients were interviewed about clinical symptoms, travel history, and exposures to food, water, and animals in the 7 days before illness onset. Completed questionnaires were returned to the Danish Zoonosis Centre and linked with microbiologic data.

All isolates included in the study were tested for resistance to nalidixic acid and erythromycin. All human isolates from the county of Copenhagen and isolates obtained from retail food products and broiler chickens were screened by a disk-diffusion test using Oxoid disks on 5% blood agar plates. On the basis of zone sizes, this method grouped the isolates in two well-separated populations of susceptible and resistant isolates with both antimicrobial drugs. The few isolates that fell between these populations were retested by using the standardized tablet diffusion and E-test procedures described previously (*5*), with the modifications that resistance to nalidixic acid was defined as an MIC >64 mg/L for the MIC method and a zone size <27 mm for the tablet method. All human isolates from the county of Funen were tested by the standardized tablet diffusion test with both antimicrobial drugs. Finally, all isolates found to be resistant and sensitive to nalidixic acid from our case-comparison study were retested by both the standardized tablet diffusion and E-test procedure.

### Case-Comparison Study

In the second half of the study period (from December 1, 2001, to June 10, 2002), characteristics of patients with quinolone-resistant and quinolone-sensitive *C. jejuni* infections were compared. Each patient with a resistant isolate was matched with two randomly selected patients with sensitive isolates. Patients were matched on date of specimen collection.

Patients answered, either by phone or by mail, a short additional questionnaire, which included questions about use of fluoroquinolones the month before onset of illness, use of fluoroquinolones after onset of illness but before specimen collection, use of antimicrobial drugs after specimen collection, and other clinical information. When patients could not answer questions about exposure to fluoroquinolones before fecal sampling, the information was gathered from their healthcare providers.

### Food and Animal Isolates

As part of a national surveillance program, food samples from retail outlet stores were analyzed for *Campylobacter* at the regional food safety authorities, according to accredited methods of the Nordic Committee on Food Analysis (*6*). The samples were taken from whole poultry and different cuts of poultry (frozen and fresh), including chicken and turkey. Samples of pork and beef products were also analyzed. Imported as well as domestic food products were sampled.

As part of a national surveillance program for *Campylobacter* in broiler chickens, chickens were sampled at slaughter and analyzed for *Campylobacter*. In this study, isolates from broiler chicken farms located in Funen County were included (one isolate per flock). Copenhagen County does not have any broiler chicken farms.

### Serotyping and Molecular Subtyping of *Campylobacter* Isolates

One isolate from the primary isolation on modified charcoal cefoperazone deoxycholate agar (mCCDA) from each patient, as well as one isolate from each retail food sample and broiler chicken fecal sample were characterized at Statens Serum Institut and the Danish Veterinary Institute. Speciation, serotyping, and RiboPrinting (automated ribotyping) were undertaken as previously described (*7**,**8*), with the following modifications for the RiboPrinting method: 1-µL eye needle was filled with bacterial culture and dissolved in 100 µL sample buffer. Ten microliters of 10 g/L lysozyme was added, and the solution was left at 37°C for 10 min. From this solution, 30 µL was transferred to a sample carrier for heat treatment. The RiboPrinter was run according to the SEC protocol at 37°C for 2 h.

### Statistical Analysis

Conditional logistic regression was applied to calculate a matched odds ratio for the exposure variables. Variables, which reached a significance level of <0.15 in the univariate analysis of the case comparison study, were selected for the multiple logistic regression analysis. Stepwise conditional logistic regression with a backward elimination procedure was conducted to obtain a reduced model. Variables with a p value <0.05 were kept in the model. All excluded variables were retested in the final model. The statistical software SAS Release v.8.00 (SAS Institute Inc., Cary, NC) and Epi Info version 6.04d (Centers for Disease Control and Prevention, Atlanta, GA) were used to analyze the data.

## Results

### Surveillance and Resistance

Of 975 culture-confirmed *Campylobacter* infections in the study, 177 (18.2%) were infected with a quinolone-resistant isolate, whereas 3 (0.3%) isolates were erythromycin-resistant. Linked microbiologic and epidemiologic data were obtained from 678 (69.5%) patients. In total, 152 (22.4%) patients had been outside Denmark within 1 week before illness, whereas 526 (77.6%) were domestically acquired infections. The three erythromycin-resistant isolates were all *C. coli*, two of them were also quinolone-resistant, and these were both isolated from travelers returning to Denmark from Spain and Portugal, respectively.

Quinolone resistance was significantly associated with the origin of infection: 76 (50.0%) of 152 infections among travelers returning to Denmark were quinolone-resistant whereas 52 (9.9%) of 526 of domestically infected patients were infected with a quinolone-resistant strain (p < 0.001) (Table 1). For both *C. coli* and *C. jejuni*, a significantly higher proportion of quinolone-resistant infections was found among patients who had been abroad in the week before onset of illness than among patients with domestically acquired infections (risk ratio [RR] 9.3, 95% confidence interval [CI] 1.4 to 63.8, p = 0.004 and RR 4.9, CI 3.6 to 6.7, p < 0.001). A higher proportion of *C. coli* than *C. jejuni* infections were acquired abroad (48.3% compared with 21.3% of *C. jejuni*).

**Table 1:** Quinolone resistance by history of recent foreign travel and comparison with *Campylobacter* isolates from food products and broiler chickens

Species	Total no.	Human (n = 678)				Food (n = 180)		Broiler chickens (n = 49)	
		Travel (n = 152)		Domestic (n = 526)					
		No.	% resistant^a^	No.	% resistant^a^	No.	% resistant^a^	No.	% resistant^a^
*C. jejuni*	1,118	137	48.2	506	9.9	153	8.5	39	5.2
*C. coli*	79	15	66.7	14	7.1	27	29.6	10	0
*C. lari*	1	0	–	1	100	0	–	0	–
*C*. spp.^b^	6	0	–	5	20	0	–	0	–
Total	1,204	152	50.0	526	9.9	180	13.7	49	5.2

^a^Quinoline-resistant isolates. ^b^Speciation not performed.

Foreign travel was associated with different prevalences of quinolone resistance, depending on destination (Table 2). No travelers returning from other Scandinavian countries hosted quinolone-resistant *Campylobacter* isolates, whereas travel to a number of regions and subregions, including southern Europe and Southeast Asia, was significantly associated with a high proportion of quinolone-resistant infections.

**Table 2:** Prevalence of quinolone resistance in *Campylobacter* isolates according to destination of foreign travel within 7 days before onset of illness^a^

Origin^b^	No. of patients	Susceptible	Resistant	% resistant	RR	95% CI	p value
Domestic (Denmark)	526	474	52	9.9	–	–	–
Southern Europe	43	15	28	65.1	6.59	4.70 to 9.24	<0.001
Northern Europe	17	17	0	0	–	–	–
Western Europe	18	10	8	44.4	4.50	2.52 to 8.01	<0.001
Central/East Europe	9	8	1	11.0	1.12	0.17 to 7.26	1.00
East Mediterranean Europe^c^	13	6	7	53.8	5.45	3.09 to 9.59	<0.001
South Asia	12	5	7	58.3	5.90	3.43 to 10.16	<0.001
Southeast Asia	13	2	11	84.6	8.56	6.05 to 12.11	<0.001
Middle East^d^	5	2	3	60.0	6.07	2.84 to 12.99	0.009
Africa	5	3	2	40.0	4.05	1.34 to 12.21	0.08
Other regions/subregions^e^	17	9	8	47.1	4.76	2.70 to 8.39	<0.001
No travel information	297	249	48	16.2	1.63	1.13 to 2.36	0.011

^a^Relative risk (RR), p value, and 95% confidence interval (CI) were calculated for the different regions/subregions with domestically acquired infections as reference. ^b^Country grouping according to the World Tourism Organization (*9*). ^c^Solely Turkey. ^d^Solely Egypt. ^e^Other regions/subregions each with less than five visits (% quinolone resistance): Australasia 1 (0); Caribbean 1 (0); North America 1 (0), South America 2 (100); North Asia 2 (50); unknown destination 1 (0); multiple subregions/regions 9 (56).

*C. jejuni* infections and *C. coli* infections did not differ in severity, as assessed by frequency of diarrhea, blood in stool, abdominal pain, fever, vomiting, mean duration of illness, or admission to hospitals. However, the mean duration of illness was longer for the 86 patients with quinolone-resistant *C. jejuni* infections and a known duration of illness (median 13.2 days) than for the 381 patients with quinolone-sensitive *C. jejuni* infections and a known duration of illness (median 10.3 days, p = 0.001). The association with extended length of illness was independent of foreign travel. For domestically acquired infections, the mean duration of illness was 12.4 and 10.4 days for quinolone-resistant and quinolone-sensitive infections, respectively. For comparison, the mean duration of illness for travel-associated infections was 13.9 and 10.3 days for quinolone-resistant and quinolone-sensitive infections, respectively. For *C. coli*, no difference in mean duration of illness was observed between quinolone-resistant and quinolone-sensitive infections.

### Case-Comparison Study

From December 1, 2001, to June 10, 2002, 42 patients were infected by quinolone-resistant *C. jejuni* isolates, and these patients were matched with 84 patients with quinolone-sensitive isolates. No patients were connected on epidemiologic grounds of a recognized outbreak. The patients with quinolone-resistant isolates had a mean age of 33 years (interquartile range 20–45 years), and a male-to-female ratio of 1:1.2. For comparison, patients with quinolone-resistant isolates in the larger study, which were not included in the case-comparison study, had a mean age of 31 years (interquartile range 20–45 years), and a male-to-female ratio of 1:1.3. No strains changed susceptibility category after being retested. However, one case-patient was shown to be co-infected with two *C. jejuni* strains with identical serotype and RiboGroup, but with different susceptibility patterns, i.e., one strain was clearly sensitive (MIC of quinolone = 2 mg/L), whereas the MIC of quinolone for the other one was 128 to >256 mg/L on multiple repeated testings. Subsequent nucleotide sequence analysis indicated a normal consensus sequence in the former and a Thr-86 to Ala-86 mutation and three silent mutations in *gyr*A in the latter. The patient had not been exposed to fluoroquinolones before stool specimen collection.

Risk factors for a quinolone-resistant *C. jejuni* infection identified in the univariate analysis and in the multiple logistic regression analysis are presented in Table 3. According to the multiple logistic regression analysis, the only exposures independently associated with an increased risk for quinolone-resistant *C. jejuni* infection were foreign travel (OR = 16.81), eating fresh poultry other than chicken and turkey (OR = 19.10), and swimming (OR = 5.01). Eating fresh chicken (of presumably Danish origin) was associated with a decreased risk (OR = 0.04). Age group did not affect the findings (younger or older than 15 years of age) in either the univariate or the multiple logistic regression analysis.

**Table 3:** Risk factors for infection with quinolone-resistant *Campylobacter jejuni* as compared with those for quinolone-sensitive *C. jejuni*^a^

Exposures	Patients with resistant isolates (n = 42)		Patients with sensitive isolates (n = 84)		Univariate analysis			Multivariate analysis		
					mOR	95% CI	p value	mOR	95% CI	p value
	No.	(%)	No.	(%)						
Travel abroad within last 7 days	30	(71.4)	12	(14.3)	12.12	4.23 to 34.73	<0.0001	16.81	3.44 to 82.20	0.001
Fluoroquinolone treatment after illness onset but before stool sample or 4 weeks before symptom onset	8	(19.1)	5	(6.0)	4.44	1.15 to 17.09	0.031	–	–	–
Beef (not cold cuts)	27	(64.3)	73	(86.9)	0.31	0.13 to 0.73	0.008	–	–	–
Fresh chicken	14	(33.3)	58	(69.6)	0.17	0.06 to 0.45	0.0004	0.04	0.004 to 0.39	0.005
Fresh poultry other than chicken and turkey	7	(16.7)	7	(8.3)	2.40	0.73 to 7.86	0.148	19.10	2.18 to 167.30	0.008
Sausages	8	(19.1)	33	(39.3)	0.32	0.12 to 0.88	0.027	–	–	–
Handling of raw meat	9	(21.4)	43	(51.2)	0.14	0.04 to 0.48	0.002	–	–	–
Public water supply	19	(45.2)	66	(78.6)	0.17	0.06 to 0.46	0.001	–	–	–
Swimming (pool, ocean, lake, or other places)	20	(47.6)	16	(19.1)	3.22	1.48 to 7.00	0.003	5.01	1.14 to 21.99	0.033
Animal contact	14	(33.3)	45	(53.6)	0.44	0.20 to 0.94	0.032	–	–	–

^a^Matched odds ratio–univariate and multivariate analysis. mOR, matched odds ratio; CI, confidence interval.

The case-comparison study identified 12 quinolone-resistant cases that were domestically acquired. However, to determine the sources of infection for the domestically acquired quinolone-resistant infections, an unmatched subanalysis on domestically acquired infections (quinolone-resistant versus quinolone-sensitive) was performed. Infections treated with fluoroquinolones before specimen collection were excluded. In this model, the parameter estimates did not change substantially from the primary model, but because of the lower sample size, the confidence intervals increased, and only eating fresh poultry other than chicken and turkey had p value <0.05. In 10 (11.9%) of 84 domestically acquired infections, patients reported eating fresh poultry other than chicken and turkey compared with 4 (9.5%) of 42 infections in persons with travel-related infections.

Overall, we found information on antimicrobial drugs received by 122 of 126 patients. Forty patients (32.8%) were treated with antimicrobial agents for their campylobacteriosis; of these, 33 patients (27%) received a fluoroquinolone, 6 patients (4.9%) received a macrolide, and 1 patient (1%) received both a fluoroquinolone and a macrolide for the *C. jejuni* infection.

### *Campylobacter* Isolates from Retail Food Products and Broiler Chickens

The human isolates were included in a database and compared with 180 *Campylobacter* isolates obtained from retail food products (chicken [n = 139], turkey [n = 39], and pork [n = 2]) and 49 isolates from broiler chicken fecal samples obtained from the same geographic area and time period as the human isolates. Most (63%) food isolates were from Danish-bred food animals; the remaining isolates were from imported food from France (n = 48), Italy (n = 7), and the United Kingdom (n = 9). The origin of three chicken isolates was unknown. Of 180 isolates obtained from food products of both domestic and foreign origin, 153 (85%) isolates and 27 (15%) isolates were *C. jejuni* and *C. coli*, respectively (Table 1). Thirteen (8.5%) of 153 *C. jejuni* isolates and 8 (29.6%) of 27 *C. coli* isolates were resistant to nalidixic acid. Three (2.0%) of 153 *C. jejuni* isolates and 5 (18.5%) of 27 *C. coli* isolates were resistant to erythromycin. Two isolates (one *C. jejuni* and one *C. coli*) from domestic chicken products were resistant to both antimicrobial agents. A subanalysis of resistance status by origin of 139 retail chicken products (domestic versus imported) showed that 7 (8.0%) of 87 *C. jejuni* isolates and three (60%) of five *C. coli* isolates from domestic raised chicken products were resistant to nalidixic acid. Of isolates from imported chicken products, 5 (14.7%) (3 isolates from France and 2 isolates from the United Kingdom) of 34 *C. jejuni* isolates and 1 (10%) (from France) of 10 *C. coli* isolates were resistant to nalidixic acid.

Of 49 isolates from broiler chicken fecal samples, 39 (79.6%) were *C. jejuni* and 10 (20.4%) were *C. coli* (Table 1). Two isolates (4.1%) (both *C. jejuni*) were nalidixic acid–resistant; one was also erythromycin-resistant. Five (10.2%) isolates (four *C. jejuni*, one *C. coli*) were erythromycin-resistant.

### Serotyping and Molecular Subtyping of Isolates

We found 133 combinations of serotypes and RiboGroups (hereafter subtypes) among 496 typed isolates (10 isolates were not tested or nontypeable) from domestically acquired *C. jejuni* infections (Table 4). Eighteen (13.5%) subtypes were identified exclusively among quinolone-resistant isolates, 102 (76.7%), exclusively among quinolone-sensitive isolates, and 13 (9.8%) among both resistant and sensitive isolates.

**Table 4:** Number of *Campylobacter jejuni* subtypes by quinolone susceptibility from domestically acquired infections, retail food products, and broiler chickens

Origin^a^	Total no. subtypes	Quinolone-resistant (%)	Quinolone-sensitive (%)	Quinolone-resistant and quinolone-sensitive (%)
Humans (n = 496)	133	18 (13.5)	102 (76.7)	13 (9.8)
Retail food products (n = 172)	81	9 (11.1)	70 (86.4)	2 (2.5)
Broiler chickens (n = 46)	20	2 (10.0)	18 (90.0)	0
Total	234	29 (12.4)	190 (81.2)	15 (6.4)

^a^Ten, 8, and 3 isolates from humans, retail food products, and broiler chickens, respectively, were not tested or nontypeable.

Five of 11 subtypes of quinolone-resistant *C. jejuni* found among isolates from retail food products, broiler chickens, or both were also found among quinolone-resistant domestically acquired *C. jejuni* isolates from humans, and 34 of 88 subtypes of quinolone-sensitive *C. jejuni* found among isolates from retail food products, broiler chickens, or both were also found among quinolone-sensitive domestically acquired *C. jejuni* isolates from humans.

Patients with domestically acquired quinolone-sensitive *C. jejuni* infections were more likely to have a *C. jejuni* subtype that was also identified among retail food products and broiler chickens than were patients with domestically acquired quinolone-resistant infections (270 of 444 vs. 15 of 51, RR = 2.07, CI 1.34 to 3.18, p < 0.001).

## Discussion

Several studies have proposed a causal relation between the veterinary use of fluoroquinolones in food production and the increase in quinolone-resistant *Campylobacter* infections in humans (*10**–**14*). However, it has been argued that use of fluoroquinolones in human medicine may be driving the increasing quinolone resistance among human *Campylobacter* isolates (*15**,**16*). Our study provides additional epidemiologic and microbiologic data to this discussion.

Our case-comparison study identified three factors to be independently associated with increased risk of attracting a quinolone-resistant *C. jejuni* infection: foreign travel, eating fresh poultry other than chicken and turkey, and swimming. Eating fresh chicken was associated with a decreased risk.

A travel association for quinolone-resistant *Campylobacter* infection has been reported from numerous countries in recent years (*14**,**17**–**20*). A limitation of most studies, apart from the Minnesota study (*14*), is that the epidemiologic information did not include a question on current or recent treatment with fluoroquinolones before stool-specimen collection. Therefore, uncontrolled confounding might have occurred. In our study, treatment with a fluoroquinolone before stool-specimen collection and having a quinolone-resistant *C. jejuni* infection, though statistically significant in the univariate analysis, was no longer significant in the multivariate analysis (Table 3). This finding suggests that quinolone use in humans is not the major selective force for quinolone resistance among *Campylobacter* spp. that cause human infections. However, use of fluoroquinolones in human medicine may still, to some degree, contribute to quinolone resistance in *Campylobacter*. This finding was instructively illustrated in our study: by an error, two strains were recovered from one *Campylobacter* episode, a fluoroquinolone-sensitive strain obtained from the patient's stool sample on December 7, 2001, and one resistant strain (MIC >256 mg/L) from the same patient's stool sample on December 14, 2001. The second stool specimen was obtained after at least 3 days' treatment with ciprofloxacin. The isolates had the same serotype and RiboGroup, and subsequent sequence analysis showed a Thr-86 to Ile-86 mutation in *gyr*A, the most common identified mutation in quinolone-resistant *C. jejuni* field strains. Treatment with quinolones has previously been shown to be associated with isolation of a resistant strain (*15**,**21**–**23*), but this case is, to our knowledge, the first documented clinical case in which the exact mutation is presented by a comparison of pre- and posttreatment *gyr*A genes. In the study by Smith et al. in Minnesota (*14*), human exposure to a fluoroquinolone before stool specimen collection was identified as a risk factor for quinolone-resistant *C. jejuni* infection, but their study also showed that treatment with a fluoroquinolone before stool culture accounted for a maximum of 15% of resistant isolates in Minnesota during 1996 and 1998. Therefore, fluoroquinolone use in humans can (and did in a small extent in this study) result in emergence of quinolone resistance in the treated patient, but the treated patient is unlikely to be a source of quinolone-resistant *Campylobacter* for other people, because person-to-person transmission of *Campylobacter* is not considered epidemiologically important.

In our study, eating fresh poultry other than chicken and turkey was rare, but a significant risk factor for both quinolone-resistant infections in general, and for domestically acquired quinolone-resistant infections. The type of fresh poultry other than chicken and turkey was not specified in the questionnaire but could have been duck, goose, or ostrich.

Swimming was also associated with an increased risk for quinolone-resistant infections. The exposure was frequently reported by travel-related infections (20 [48%] of 42), compared with domestically acquired infections (16 [19%] of 84). Patients were questioned about swimming in pool, ocean, lake, or other places combined. Future studies should specify the type of water more specifically.

Eating fresh chicken was associated with a decreased risk for quinolone-resistant infections. The fresh chicken was of presumably Danish origin, as most fresh chicken eaten in Denmark is domestically raised. In addition, as travelers often eat at restaurants, where information about whether a served chicken is fresh or has been frozen is normally not available, patients who reported eating fresh chicken were likely to have consumed it in Denmark. This is supported by the fact that of 56 (67%) of 84 domestically acquired infections, patients reported eating fresh poultry compared with 17 (38%) of 42 patients with travel-related infections. Eating poultry is believed to be the primary means of acquiring human campylobacteriosis, although other sources also exist (*1*). This corroborates the hypothesis that quinolone-resistant–*C. jejuni* infections could result from the use of quinolones in animals and in food production. The veterinary antimicrobial drug use hypothesis is supported by the findings of this study and studies by our European and American colleagues that a significantly higher proportion of quinolone-resistant *C. jejuni* infections occur among patients who had been abroad, often to destinations with recognized high quinolone-resistance in *Campylobacter* in food animals as well as established high risk of attracting quinolone-resistant human *Campylobacter* infections, than among domestically acquired infections (Tables 1 and 2) (*3**,**11**,**12**,**18**,**24**–**27*). In Denmark, as part of the Danish Integrated Antimicrobial Resistance Monitoring Programme (DANMAP), antimicrobial drug susceptibility in *Campylobacter* is monitored systematically in food animals, retail food products, and humans as well as use of antimicrobial drugs, including quinolones at food animal species level. Compared to the practice in many other countries, only small amounts of fluoroquinolones are used in broiler chicken production, and during 2002, use of fluoroquinolones decreased significantly after restrictions imposed by the Danish Veterinary and Food Administration called for reducing fluoroquinolone use (*28*). According to DANMAP surveillance data for 2002, no resistance among *C. jejuni* to quinolones was found in broiler chickens, and quinolone resistance to *C. jejuni* was found in only 6% of imported and domestic retail chicken meat (*28*). This finding may explain why eating fresh chicken (of presumably Danish origin) was associated with a decreased risk for quinolone-resistant *C. jejuni* infection in the matched multivariate analysis. Our typing data also support this explanation, because patients with domestically acquired quinolone-sensitive *C. jejuni* infections were more likely to have a *C. jejuni* subtype that was also identified among retail food products and broiler chickens than were patients with domestically acquired quinolone-resistant infections.

A potential limitation of our study is the fact that only one isolate from each retail food sample or broiler fecal sample was characterized. Previous studies have shown that multiple strains of *Campylobacter* may be recovered. Capturing the diversity of strains may have been helpful in accounting for a higher percentage of human strains, as would analysis of additional food and broiler chicken samples.

In many countries, including Denmark, fewer *Campylobacter* infections are identified in the winter months, but among the ones that are, a higher percentage are associated with foreign travel. In the case-comparison study, 30 (71%) of 42 quinolone-resistant *C. jejuni* infections were associated with foreign travel versus 66 (57%) of 116 quinolone-resistant *C. jejuni* infections in the larger study (not seasonal). A limitation of our study is therefore its time frame (December–June). The patients with resistant isolates in the case-comparison study were demographically (age and sex) comparable to the group of quinolone-resistant infections from the larger study, which were not included in the case-comparison study.

In the Minnesota study (*14*), a clinical implication of fluoroquinolone-resistance among *C. jejuni* infections was identified: the duration of diarrhea among patients treated with a fluoroquinolone was significantly longer if the patient had a fluoroquinolone-resistant infection (median 10 days) versus a fluoroquinolone-susceptible infection (median 7 days). We also found significantly longer duration of illness among patients with quinolone-resistant *C. jejuni* infections (median 13.2 days) compared to that of patients with quinolone-sensitive infections (median 10.3 days). However, as history of antimicrobial treatment was only obtained from the case-comparison proportion of our study, stratifying by treatment to determine whether the negative impact on public health was caused by true treatment failures is not possible.

We found three macrolide-resistant strains; all were *C. coli* isolated from travelers returning to Denmark. Our finding is in line with current surveillance data on the level of macrolide resistance in Danish broiler chickens, cattle, and chicken meat (*28*). Resistance to macrolides has also been reported at continued low level in a number of other countries and should remain the first drug of choice for verified campylobacteriosis (*3**,**29*).

In conclusion, the current study found evidence of prolonged duration of illness associated with quinolone resistance and supports the conclusions drawn by the U.S. Food and Drug Administration: human quinolone-resistant *Campylobacter* infections have increased, and this increase has a negative impact on public health. This study also suggests that in a country with restricted fluoroquinolone use in poultry production, chicken is not a source of domestically acquired quinolone-resistant *Campylobacter* infections, and that in countries with less restrictive use, poultry is an important source of such infections. The use of fluoroquinolones for food production animals should be discontinued or minimized to preserve fluoroquinolone sensitivity in *Campylobacter*.
